# The Neuropsychiatric Changes After COVID-19 Quarantine in Patients With Cognitive Impairment and Their Caregivers in Chongqing, China: A Cohort Study

**DOI:** 10.3389/fnagi.2021.762907

**Published:** 2022-02-10

**Authors:** Shiyun Yuan, Wenbo Zhang, Qiang Yao, Wenqi Lü, Wuhan Yu, Fuxin Zhong, Yan Wang, Dianxia Xing, Xiaoqin Wang, Jiaqi Song, Hong Huang, Chenxi Chen, Junjin Liu, Weihua Yu, Yang Lü

**Affiliations:** ^1^Department of Geriatrics, The First Affiliated Hospital of Chongqing Medical University, Chongqing, China; ^2^Department of Epidemiology and Health Statistics, West China School of Public Health and West China Fourth Hospital, Sichuan University, Sichuan, China; ^3^Department of Psychiatry, West China Hospital, Sichuan University, Sichuan, China; ^4^Institutes of Neuroscience, Chongqing Medical University, Chongqing, China; ^5^Foreign Language College, Chongqing Medical University, Chongqing, China

**Keywords:** COVID-19, neuropsychiatric impact, cognitive impairment, caregivers, cohort study

## Abstract

**Background:**

The follow-up study on neuropsychiatric changes after the lifting of coronavirus disease 2019 (COVID-19) quarantine in patients with cognitive impairment and their caregivers is still lacking, and relative information is needed to formulate more comprehensive healthcare prevention measures worldwide.

**Aims:**

To provide data on the changes in neuropsychiatric performance after the lifting of COVID-19 quarantine in patients with cognitive disorders and their caregivers.

**Methods:**

Two surveys in Chongqing, China were conducted *via* telephonic interview with 531 patients and their caregivers. The baseline survey was performed from February 11 to 23, 2020, and the follow-up was from October 24 to November 9, 2020. The data of neuropsychiatric symptoms (NPSs), sleep, nutrition, and chronic diseases of patients, as well as the burden of care, anxiety, and depression of caregivers were evaluated.

**Results:**

Significant alleviation of NPSs after the lifting of COVID-19 quarantine was observed in patients with mild cognitive impairment (MCI) and dementia (both *P* < 0.05). Compared with baseline, the prevalence for NPSs of all participants dropped from 57.94 to 38.82%. Among NPS subdomains, apathy displayed the biggest decline at follow-up by 10.72%, followed by nighttime behavior by 8.65%. Mixed effect generalized estimation equation analysis showed significant amelioration in hallucination, depression, apathy, irritability, aberrant motor behavior, and nighttime behavior (all *P* < 0.05), with the most prominent changes in nighttime behavior and apathy. Among the patients with unsatisfactory control of chronic disease, the medication adherence rate dropped by approximately 30% after the lifting of quarantine. More importantly, around 13% increase of care burden was observed among the caregivers at follow-up, with both depression and anxiety rising by nearly 4%.

**Conclusion:**

The prolonged quarantine may exacerbate NPS in patients with memory disorders, while the care burden and mental stability of the caregivers after the pandemic should also be concerned.

## Introduction

Unprecedented large-scale quarantine measures have been implemented worldwide, including China, to fight against a novel coronavirus disease 2019 (COVID-19) since 2019. This intense situation is likely to have a negative psychosocial effect on residents (Brooks et al., 2020; Wu and McGoogan, 2020; Vrillon et al., 2021; World Health Organization, 2021). As a vulnerable population, patients with dementia, including all-cause dementia and Alzheimer’s disease (AD), suffered an increased risk of COVID-19-related death (Fox and Petersen, 2013; Alzheimer’s Disease International World Alzheimer’s report 2019, 2021; Zhu et al., 2021) due to their low cognitive ability and poor awareness of risk (Alonso-Lana et al., 2020; China Daily, 2020; Esposito, 2020; Shanxi Provincial Center for Disease Control and Prevention, 2020; Williamson et al., 2020; Yao et al., 2020).

Coherent studies on the status of patients with dementia or memory disorders were carried out in countries worldwide, such as India, Spain, the United States, and Argentina. However, most of these studies adopted semi-structured interviews or self-designed questionnaires (Cohen et al., 2020; Goodman-Casanova et al., 2020; Rajagopalan et al., 2021; Rising et al., 2021) that may lack wide applicability and representativeness. In one of our previous studies, we interviewed 787 patients and their caregivers using widely used assessment scales such as neuropsychiatric inventory (NPI), Patient Health Questionnaire-9 (PHQ-9), Generalized Anxiety Disorder Scale (GAD-7), and Relative Stress Scale (RSS). We discovered that neuropsychiatric symptoms (NPSs), such as depression and anxiety, aggravated in patients with memory disorders and their caregivers during COVID-19, and medication adherence contributed to the stabilization of NPSs (Yuan et al., 2021).

The impacts of traumatic events or disasters often persist long, and survivors may develop delayed-type post-traumatic stress disorder (Chou et al., 2007; Nifenecker, 2015; Loganovsky et al., 2020). As a global public health event that has lasted for more than one and a half years, the COVID-19 pandemic attracts great attention for its subsequent impact on humans (Ismail et al., 2021). However, currently, few studies have been reported on this after-pandemic impact since the disease is still spreading.

On April 8, 2020, China periodically contained the epidemic and transited to the recovery of both economy and social life. Data on the changes in the psychological status of patients with cognitive impairment and of their care burden provide important information for preventative healthcare planning. These countermeasures could be beneficial for patients as well as for caregivers to reduce mental problems. Therefore, we conducted the follow-up interview in the same population who have participated in our first survey during the quarantine (Yuan et al., 2021). In the follow-up investigation, the same questionnaires were applied during October 24 and November 9, 2020, regarding COVID-19 contact history, NPSs, sleep quality, nutrition of patients, as well as care burden and mental stress of their caregivers. We hope our work would provide a reference for healthcare for other countries when they experience the recovery from the COVID-19 pandemic.

## Materials and Methods

### Participants

Notably, 531 out of 787 patients who participated in our first investigation (Yuan et al., 2021) completed the follow-up interview *via* telephone from October 24 to November 9, 2020, at 6 months after the national quarantine was lifted. The reasons for lost patients (*N* = 256) in the follow up were out of service (*N* = 27), failed connection (*N* = 55), and refused to participate (*N* = 174). The interview contained the following five aspects: (1) demographic information; (2) exposure history of COVID-19; (3) medication, chronic disease management, evaluation of nutrient status, and sleep quality; (4) NPSs of patients; and (5) the care burden, anxiety, and depression of caregivers. This study was approved by the Ethics Committee of The First Affiliated Hospital of Chongqing Medical University (Certificate No. 20200301).

### Instruments

The scales applied in this study were consistent with our first investigation (Yuan et al., 2021), including NPI, PHQ-9, GDS-7, RSS, Mini Nutritional Assessment Short Form (MNA-SF), and Pittsburgh Sleep Quality Index (PSQI). The investigation process and rating were conducted as described before (Brooks et al., 2020). For MNA-SF in this study, the at-risk of malnutrition and malnutrition were defined as nutritional deficiency. All participants contacted at follow-up were in the same order as at baseline.

### Statistical Analyses

Continuous measurements are presented as *Mean* ± *SD* if they are normally distributed, or *Median* with Q1 and Q3 if not, and categorical variables as count and frequency (%). All statistical analyses were performed using SPSS 26.0 (IBM, Armonk, NY, United States). Demographic characteristics were evaluated by frequency distributions. The comparison of all scale performances between baseline and follow-up was analyzed using the mixed effect generalized estimation equation, with age, sex, and diagnosis of the patient as covariates. The changes in the nutritional status of patients, including food intake and body weight, and the changes in the prevalence of care burden, depression, and anxiety of caregivers were analyzed using the chi-square test.

## Results

### General Information

A final number of 531 respondents completed both baseline and follow-up investigations, with 182 (34.27%) men and 349 (65.72%) women at the median age of 74.3 years (Table 1). The proportion of each diagnosis is shown in Supplementary Figure 1, in which AD took up the biggest proportion (45.39%). Then, subjects were regrouped by diagnoses with mild cognitive impairment (MCI) (*N* = 158, 29.76%), AD (*N* = 241, 45.39%), non-AD dementia (*N* = 51, 9.60%), and other diseases (*N* = 81, 15.25%). The outcomes of each scale at both baseline and follow-up were presented by median and IQR in total and in each group. Primarily, the median of NPI for all patients at follow-up, as compared with baseline, dropped by 1 point, with a reduction by 3, 4, and 1 point in groups of AD, non-AD dementia, and other diseases, respectively. Consistently, the median of NPI distress for caregivers of both patients with AD and non-AD dementia decreased by 2 points; however, in these two groups, the median of RSS results for caregivers increased at follow-up by 2.5 and 3 points, respectively.

**TABLE 1:** Demographic and general evaluative outcomes of participants enrolled in the cohort study.

	Total (*n* = 531)		MCI (*n* = 158)		AD (*n* = 241)		Non-AD Dementia (*n* = 51)		Other diseases (*n* = 81)	
**Sex(n,%)**										
Male	182 (34.3%)		57 (36.1%)		80 (33.2%)		21 (41.2%)		24 (29.6%)	
Female	349 (65.7%)		101 (63.9%)		161 (66.8%)		30 (48.8%)		57 (70.4%)	
Age	74.33 ± 9.693		72.65 ± 9.521		77.00 ± 8.474		78.27 ± 7.713		67.17 ± 9.812	
Time	Baseline	Follow-up	Baseline	Follow-up	Baseline	Follow-up	Baseline	Follow-up	Baseline	Follow-up
NPI	2(0-9)	0(0-5)	0(0-4)	0(0-2.25)	4(0-12)	1(0-8)	4(0-11)	0(0-5)	1(0-5)	0(0-3)
NPI-D	0(0-4)	0(0-2)	0(0-2)	0(0-1)	2(0-6)	0(0-3)	2(0-6)	0(0-3)	0(0-2)	0(0-0.5)
MNA-SF	11 (10-12)	11 (9-13)	12 (10-13)	11 (9-12)	11 (9-12)	11 (9-12)	11 (9-12)	11 (9-12)	12 (9-13)	12 (9-13)
PSQI*	4(2-7)	4(2-7)	4(2-7)	4(2-8)	4(2-7)	4(2-7)	4(2-7)	4(2-7)	5(2-8)	4(1.5-7)
RSS*	0.5(0-7)	0(0-6)	0(0-1)	0(0-1)	0(0-2)	2.5(0-10)	0(0-2)	3(0-11)	0(0-2)	0(0-2)
PHQ-9*	0(0-1)	0(0-0.25)	0(0-0)	0(0-0)	0(0-1)	0(0-1.75)	0(0-0)	0(0-1)	0(0-0)	0(0-0)
GAD-7*	0(0-0)	0(0-0)	0(0-0)	0(0-0)	0(0-0)	0(0-1)	0(0-0)	0(0-1)	0(0-0)	0(0-0)

*Baseline: From February 11, 2020 to February 23, 2020, 1 month after national quarantine implemented. Follow-up: from October 19, 2020 to November 9, 2020, 8 months after national quarantine was lifted. Age was portrayed by mean ± SD, and the scale values were presented by medians and interquartile ranges categorized by diagnosis both at baseline and follow-up.*

*MCI, Mild Cognitive Impairment; AD, Alzheimer’s Disease; NPI, Neuropsychiatric Inventory; NPI-D, Caregiver Distress; MNA-SF, Mini Nutritional Assessment Short Form; PSQI, Pittsburgh Sleep Quality Index; RSS, Relative Stress Scale; PHQ-9, Patient Health Questionnaire-9; GAD-7, Generalized Anxiety Disorder Scale.*

*The subjects for RSS, PHQ-9, and GAD-7 were all caregivers.*

*Due to incomplete data, 15 cases in PSQI were excluded in the baseline survey, while 3 cases were excluded in the follow-up survey. 19 cases in RSS were excluded in the baseline survey due to having no caregivers. A total of 14 were excluded in both PHQ-9 and GAD-7 in baseline and 1 case was excluded in the follow-up survey due to having no caregivers.

### COVID-19-Related Data

In the baseline survey, 68 patients reported their worries about the outbreak, while this number reduced to 12 after 8 months. None of the participants reported contact history with people from medium/high-risk areas in the follow-up investigation. Neither the subjects nor their families were diagnosed or suspected with COVID-19 at baseline and follow-up (Table 2).

**TABLE 2:** Exposure history and worrisome to COVID-19 outbreak and quarantine.

Baseline (n,%)		Follow-up (n,%)	
Contact history with people from Wuhan or Hubei	4(0.51%)	Contact history with people from medium-high risk areas in China or abroad	0 (0.00%)
Domestic or overseas traveling experience within 2 weeks	3(0.38%)	Domestic medium-high risk area or overseas traveling experience within 2 weeks	0 (0.00%)
Family members diagnosed or suspected as COVID-19	0(0.00%)	Family members diagnosed or suspected as COVID-19	0 (0.00%)
Worrisome for COVID-19 outbreak	68(8.61%)	Worrisome for COVID-19 outbreak	12 (2.26%)

*In the beginning of the COVID-19 outbreak in China, Wuhan was the first city to be widely affected by the outbreak. At that particular intense period of time, people who had contact history with residents in Wuhan or Hubei were regarded as having a high risk of infecting COVID-19. As are shown, only 4 patients reported a contact history with the epidemic residents and 3 had domestic or overseas traveling experience in the past 2 weeks at baseline period, while there were none at the follow-up. In the beginning of the quarantine, 8.61% of the participants were worried about the outbreak of COVID-19 while the rate declined to only 2.26% 8 months later.*

### Health-Related Status

**FIGURE 1:**
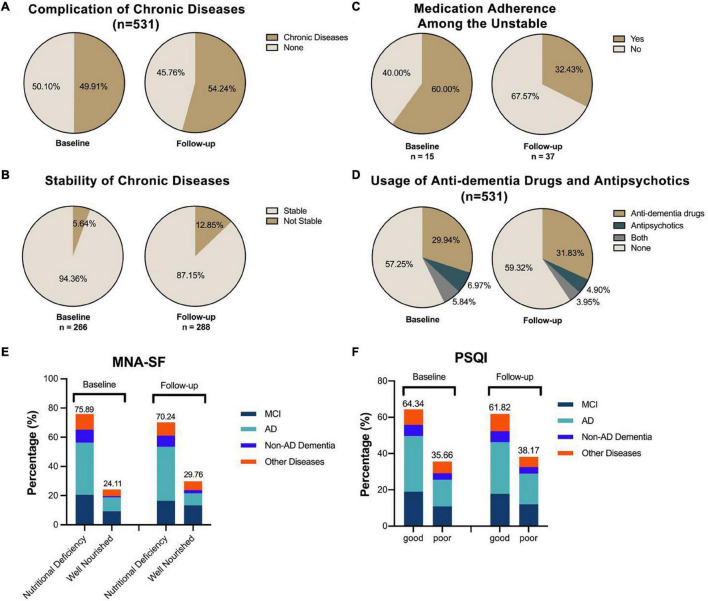
The fluctuation of chronic disease control, nutritional status, and sleep quality. The percentage of patients enrolled having chronic diseases rose nearly 4% from the baseline level **(A)**, and the instability of such disease also increased by around 7% **(B)**. Among the patients who claimed the unstable control of their chronic disease, the rate of medication adherence dropped from 60.00 to 32.43% **(C)**. The percentage of taking anti-dementia and/or antipsychotics was rising by around 7%, reaching nearly half of the subjects receiving corresponding treatment **(D)**. For nutritional status **(E)**, the at-risk-of-malnutrition and malnutrition groups were defined as nutritional deficiency. The results for the Pittsburgh Sleep Quality Index (PSQI)-based binary classification of sleep quality revealed no significant difference between these two investigations **(F)**.

As summarized in Figure 1, patients with chronic disease rose from 266 (49.91%) at baseline to 288 (54.24%) at follow-up (Figure 1A), of whom the proportion of unstable chronic disease increased by 7.21% (Figure 1B). Among the patients with unsatisfactory control of chronic disease, the rate of medication adherence dropped from 60.00 to 32.43%, by around 30% (Figure 1C). As for anti-dementia treatment, 159 (29.94%) took only anti-dementia drugs, 37 (6.97%) took antipsychotics alone, and 31 (5.84%) took both. Notably, 6 months after the lifting of quarantine, both the number and percentage of patients receiving anti-dementia treatment stayed nearly the same, with a slight drop of antipsychotic use by 2.07% (Figure 1D). Regarding the nutritional status, 75.89% of the respondents were found to be nutritionally deficient, and 24.11% were well-nourished during the quarantine; however, at follow-up, the rate of nutritional deficiency reduced to 70.24%, but with a nearly 5% rise in the well-nourished (29.76%) (Figure 1E). The results for PSQI-based binary classification of sleep quality were not much of a difference between the baseline and follow-up investigations (Figure 1F). However, in each subitem of PSQI, aggregated symptoms were observed (Supplementary Figures 2A–H). The sleep quality and sleep efficiency of patients from different diagnostic groups became worse at follow-up than that at baseline (all *P* < 0.05), and the sleep duration of patients with MCI and AD was statistically shortened at follow-up, compared with baseline (both *P* < 0.05). However, the investigation on sleep latency, sleep disorder, hypnotic usage, and daytime function revealed no significant difference among all groups (all *P* > 0.05).

### Changes in Neuropsychiatric Symptom Performances

**FIGURE 2:**
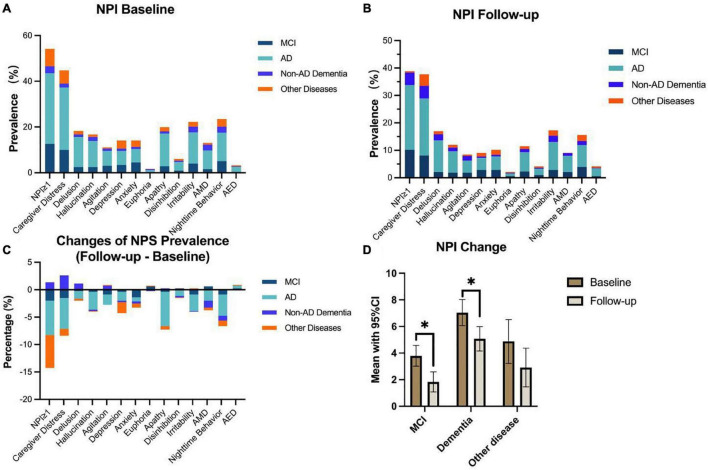
Neuropsychiatric inventory (NPI) changes among each neuropsychiatric symptom (NPS) subdomain and diagnostic group. Nearly, 55% of participants had at least one type of NPS at baseline, and this rate dropped to lower than 40% at follow-up **(A,B)**. The most prevalent NPS were nighttime behavior (23.54%), irritability (22.22%), and apathy (19.95%) at baseline **(A)**, while at follow-up, this ranking changed to irritability (17.32%), delusion (16.95%), and nighttime behavior (15.62%) **(B)**. As for the changes of NPS prevalence **(C)**, compared with baseline, nearly all NPS, except for euphoria and AED, showed a downward trend, among which apathy and nighttime behavior were the leading subdomains (dropped by 8.44% and 7.92%, respectively). In generalized estimation equation analysis, patients with MCI and dementia revealed significant alleviation in NPS performance in 8 months after the quarantine (*P < 0.05) **(D)**.

The prevalence of NPS in patients with cognitive impairment was attenuated 6 months after the lifting of COVID-19 quarantine. As presented in Figure 2, the general prevalence for NPS dropped by nearly 20%, from 57.94 to 38.82%. At baseline, nighttime behavior (24.27%) was the most prevalent NPS followed by apathy (22.24%) and irritability (21.60%), while at follow-up, irritability (17.32%) and nighttime behavior (15.62%) took the leading role, whereas apathy dropped to the third (11.51%). In Figure 2C, apathy was the most prominent symptom with the biggest decline in prevalence by 10.72%, and nighttime behavior dropped by 8.65%, ranking second.

The mixed effect generalized estimation equation was performed to total NPI scores, NPI-D, and all NPS subdomains with age, sex, and diagnosis of the patient as covariates (Table 3). The total NPI was significantly decreased in all patients (*P* < 0.001), demonstrating the improvement of NPS after the lifting of quarantine. The subdomains of hallucination, depression, apathy, irritability, aberrant motor behavior (AMB), and nighttime behavior significantly ameliorated (all *P* < 0.05), with nighttime behavior and apathy showing the greatest changes (coefficients were -0.542 and -0.429, respectively). Significant improvements in nighttime behavior, especially, were found in MCI and AD groups (both *P* < 0.05) (Supplementary Figure 2H). Figure 2D shows the results of mixed effect generalized estimation equation analysis with the intercept of diagnosis. As is shown, patients with MCI and dementia (combined AD and non-AD dementia) revealed significant alleviation in NPS performance (*P* < 0.05).

**TABLE 3:** Generalized estimation equation for mixed effects on each evaluative item.

Item	Time	Forest Plot of Coefficient	Coefficient (95% CI)	*P* Value
NPI	[Frame1]Baseline			–
		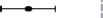		
	Follow-up		–1.968 (–2.690. –1.246)	< 0.001
NPI-D	Baseline			-
		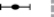		
	Follow-up		– 0.983 (–1.374, –0.593)	< 0.001
Delusion	Baseline			–
		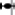		
	Follow-up		– 0.105 (–0.276, 0.065)	0.224
Hallucination	Baseline			–
		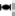		
	Follow-up		– 0.168 (–0.323, –0.012)	0.035
Agitation	Baseline			–
		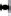		
	Follow-up		– 0.102 (–0.205, 0.002)	0.054
Depression	Baseline			–
		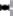		
	Follow-up		– 0.158 (–0.265, –0.051)	0.004
Anxiety	Baseline			–
		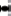		
	Follow-up		– 0.109 (–0.226, 0.007)	0.066
Euphoria	Baseline			–
		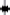		
	Follow-up		0.01 (–0.053, 0.075)	0.730
Apathy	Baseline			–
		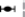		
	Follow-up		– 0.429 (–0.618, –0.241)	< 0.001
Disinhibition	Baseline			–
		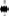		
	Follow-up		– 0.017 (–0.085, 0.051)	0.625
Irritability	Baseline			–
		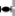		
	Follow-up		– 0.198 (–0.340, –0.055)	0.007
ABM	Baseline			–
		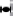		
	Follow-up		– 0.132 (–0.260, –0.003)	0.045
Nighttime Behavior	Baseline			–
		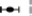		
	Follow-up		– 0.542 (–0.783, –0.301)	< 0.001
AED	Baseline			–
		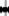		
	Follow-up		– 0.017 (–0.110, 0.076)	0.721
MNA-SF	Baseline			–
		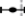		
	Follow-up		– 0.228 (–0.532, 0.076)	0.142
PSQI	Baseline			–
		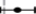		
	Follow-up		0.360 (–0.089, 0.808)	0.116
RSS	Baseline			–
		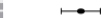		
	Follow-up		2.108 (1.583, 2.633)	< 0.001
PHQ-9	Baseline			–
		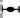		
	Follow-up		0.235 (0.016, 0.454)	0.035
GAD-7	Baseline			–
		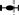		
	Follow-up		–0.105 (–0.330, 0.119)	0.358

*The subjects for RSS, PHQ-9, and GAD-7 were all caregivers.*

*The generalized estimation equation was performed to total NPI scores, NPI-D, and all NPS subdomains with the intercept of time of the investigation. For all patients, the total NPI was significantly decreased (P < 0.001), demonstrating that the improvement of NPS after domestic epidemic and quarantine. Among the 12 subdomains, nighttime behavior and apathy displayed the greatest changes (coefficients were -0.542 and -0.429, respectively, both P < 0.001). For caregivers, although NPI-D in the follow-up investigation displayed significant reduction (P < 0.001), their responses in RSS and PHQ-9 showed the opposite outcome, with higher scores in the follow-up period (coefficients were 2.108 and 0.235, respectively, both P < 0.05), implying that the caregivers had greater care burden and heavier mental issue compared with the status during the quarantine. All models were adjusted for sex, age, and diagnosis of the patient.*

### Care Burden, Anxiety, and Depression of Caregivers

**FIGURE 3:**
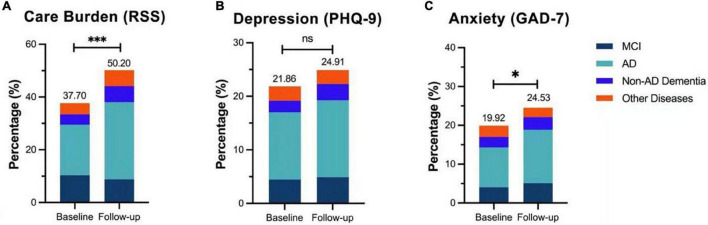
Care burden and mental status of caregivers. Notably, 50.20% of caregivers suffered from different degrees of care burden in follow-up with a nearly 13% increase from the baseline (χ^2^ = 10.293, ***P < 0.001) **(A)**. After the quarantine, the prevalence of depression among caregivers was elevated by 3.05% (χ^2^ = 1.357, *P* = 0.137) **(B)**, and anxiety increased by 4.61% (χ^2^ = 3.209, *P < 0.05) **(C)**.

We observed that the prevalence of anxiety, depression, and care burden among caregivers increased after the lifting of quarantine compared with the quarantine duration (Table 3). Notably, 50.20% of caregivers suffered from varying degrees of burden as reported in the follow-up, compared with 37.70% at the beginning of quarantine (*P* < 0.001). After the lifting of quarantine, the prevalence of depression and anxiety among caregivers was 24.91% and 24.53%, respectively, as compared with 21.86% and 19.92%, respectively, during the quarantine (Figure 3).

Mixed effect generalized estimation equation analysis (Table 3) showed that NPI-D in the follow-up investigation significantly decreased (*P* < 0.001); however, RSS and PHQ-9 increased, with higher scores in both scales (coefficients were 2.108 and 0.235, respectively, both *P* < 0.05), implying that the caregivers had greater care burden and heavier mental problems after the lifting of quarantine compared with during the quarantine, and the increased care burden was not associated with NPS performances of patients.

## Discussion

In our follow-up study, we observed that most of the NPSs were alleviated in patients with memory disorders after the pandemic; however, their caregivers suffered more from care burden and mental problems compared with the time during the quarantine. Our results indicate that these two different populations respond differently to unprecedented disasters such as COVID-19 pandemic, and post-epidemic changes may greatly affect the care burden and mental stability of caregivers. The prolonged quarantine may exacerbate NPSs in patients with memory disorders, so efficient and individual-targeted measures for better management and accompanying these patients under such circumstances are of vital significance. More importantly, the post-quarantine stress and mental problems of caregivers should be greatly concerned. To our knowledge, this is the first study to follow up the special families after the lifting of COVID-19 quarantine.

In this study, we found that the changes in the prevalence of NPSs in patients with MCI and dementia were statistically significant (both *P* < 0.05), which illustrates the significant alleviation of NPSs after the lifting of COVID-19 quarantine. Although the percentage of anti-dementia drug use in all patients at follow-up was 35.78%, the same with that at baseline, the use of antipsychotics dropped by nearly 2% after the lifting of quarantine, which was in accordance with the attenuated performance in NPSs of all patients. Our findings suggest that the large-scale, prolonged quarantine due to the pandemic may exacerbate NPSs in patients with memory disorders. The reasons for this exacerbation were the change of living habits and daily schedule during quarantine, such as confinement to relatively small spaces at home, limited exercise, and outdoor activities.

Surprisingly, we found that the prevalence of anxiety, depression, and care burden among caregivers was increased in follow-up investigation compared with baseline. Since the overall NPS performances in all patients improved at follow-up, the increased prevalence of anxiety, depression, and care burden among caregivers after the lifting of quarantine was more likely due to post-quarantine stress. All family members were not allowed to go out during the quarantine; therefore, they could share the burden of caring for patients. Caregivers were required to work outside the home in addition to caring for patients after the lifting of quarantine. This causes them to feel a heavier burden of care than during isolation. What was worse, the unemployment rate in China rose from 3.61% in the fourth quarter of 2019 to 4.24% in the fourth quarter of 2020 due to the impact of the pandemic (China’s Unemployment Rat, 2021), indicating that more and more people were under work crisis after the long period of quarantine. Consequently, caregivers might face unemployment and economic burden after the lifting of quarantine. However, after the quarantine was lifted, the caregivers returned to the routine as it used to be, which posed a very hard transition on them. Since the psychological problems of these caregivers have always been a hot issue (Vernooij-Dassen et al., 2011; Egilstrod et al., 2019) and an increased prevalence of care burden, depression and anxiety were found in this follow-up study, and we suggested that preventive measures, especially early and continuous psychiatric interventions or online self-management support (Huis In Het Veld et al., 2019), should be promoted among these family caregivers.

In this study, we observed a decrease in the number of respondents concerned about the COVID-19 pandemic after the lifting of quarantine compared with during the quarantine. This may be because, during the quarantine in March 2020, people did not know when the COVID-19 pandemic would be effectively controlled or when the quarantine would be lifted to return to normal work and life. During the after-quarantine period around November 2020, the effective control of the pandemic in China may decrease the number of respondents who are worried about the COVID-19 pandemic.

Of the 531 subjects in this study, the number of patients taking anti-dementia drugs at follow-up increased by less than 2% from baseline. This was mainly because patients who were not previously taking anti-dementia drugs got worse over time and had to start taking them. Compared with our first survey, there was an increase in the number of patients with chronic diseases after the lifting of quarantine. Furthermore, the number of patients with unstable chronic conditions is more than doubled from baseline, which is likely to be caused by failure of medication adherence. We found that the number of chronically unstable patients who did not adhere to their medication in the follow-up survey was more than four times higher than those at the baseline.

As previously reported, the medication adherence of patients may be influenced by caregiver factors, such as commitment or intention, self-efficacy, and health knowledge (El-Saifi et al., 2019), or by the cognitive impairment of patients themselves (Cho et al., 2018). In this particular situation where some caregivers were facing post-epidemic socioeconomic problems, the time and energy they spent on the patients might greatly reduce, which may also affect the medication adherence. Telehealth home monitoring and treatment modification were the only interventions reported in the literature to improve medication adherence in patients with cognitive impairment (El-Saifi et al., 2018); however, in this study, we suggested that the mental stability of caregivers should be improved by carrying out policies and assurances to reduce the rate of their unemployment after the epidemic of COVID-19 so as to effectively help with medication adherence of patients.

We observed that the number of respondents who were well-nourished after the lifting of quarantine was higher than during the quarantine. Patients were not able to enjoy outdoor activities and exercise during the quarantine, which might cause them to lose their appetite and eat less, leading to malnutrition. Similarly, we observed an increase in the number of patients with poor sleep quality during follow-up surveys. More than half of our respondents lived with and were cared for by their families. During home quarantine, families did not have to go out to work or school; therefore, they spent more time caring for patients with better quality.

There might be some kind of bias in the administration of these standardized scales through telephonic interview. However, during the nationwide lockdown, telephonic interview was the best option for this study. In addition, the scales we used were in the form of questionnaires, which had been used in telephonic interview by previous studies (Alves et al., 2020; Barguilla et al., 2020; Di Santo et al., 2020; Lara et al., 2020; Borelli et al., 2021); therefore, we administrated the scales by telephone.

## Conclusion

The prolonged quarantine may exacerbate NPSs in patients with memory disorders and pose an increased burden on caregivers. The prevalence and severity of NPSs in these patients were attenuated after the lifting of quarantine; however, the care burden and prevalence of mental problems in caregivers increased due to post-quarantine socioeconomic changes. Interventions to help improve medication adherence in adherence in patients with cognitive impairment and their caregivers should be recommended. We hope that our work can provide useful information for other countries to care for patients with cognitive impairment and their caregivers, especially, in this COVID-19 pandemic period.

## Data Availability Statement

The raw data supporting the conclusions of this article will be made available by the authors, without undue reservation.

## Ethics Statement

The studies involving human participants were reviewed and approved by Ethics Committee of The First Affiliated Hospital of Chongqing Medical University. Written informed consent to participate in this study was provided by the participants’ legal guardian/next of kin.

## Author Contributions

YL, SY, and WZ participated in the design of the research scheme. SY, WZ, WL, WeY, FZ, DX, XW, JS, CC, and JL participated in the telephonic survey and data collection. SY was responsible for the aggregation and collation of data. SY, WZ, and QY were responsible for statistics. WZ was responsible for the production of figures. SY and WZ drafted the manuscript. YW proofread and edited the manuscript to improve the language. YL revised the final manuscript. All authors reviewed, revised, and approved the final manuscript.

## Conflict of Interest

The authors declare that the research was conducted in the absence of any commercial or financial relationships that could be construed as a potential conflict of interest.

## Publisher’s Note

All claims expressed in this article are solely those of the authors and do not necessarily represent those of their affiliated organizations, or those of the publisher, the editors and the reviewers. Any product that may be evaluated in this article, or claim that may be made by its manufacturer, is not guaranteed or endorsed by the publisher.
