# Supplementary material for: The Neuropsychiatric Changes After COVID-19 Quarantine in Patients With Cognitive Impairment and Their Caregivers in Chongqing, China: A Cohort Study
Source: Front Aging Neurosci. 2022 Feb 10;13:762907. doi: 10.3389/fnagi.2021.762907 (PMC8868570; doi:10.3389/fnagi.2021.762907)
Supplement: Supplementary file 1 [file Data_Sheet_1.docx]

Supplementary Material

# Supplementary Data

**The rating rules for the scales we used in this study**

**Mini Nutritional Assessment Short Form (MNA-SF)**

MNA-SF was used to evaluate the nutritional status of the participants. As a valid and sensitive nutrition screen instrument, MNA-SF is well-suited for nutritional status assessment. In this study, participants were classified as malnutrition (MNA-SF score <8), at risk of malnutrition (MNA-SF score 8-11) or well-nourished (MNA-SF score >11) [1]. Furthermore, two additional items were added to detect appetite increase and body weight gain.

**Pittsburgh Sleep Quality Index (PSQI)**

PSQI was used to evaluate respondents' sleep quality over the past month. PSQI contains 18 items in total, and the score of each item can be divided into four levels of 0, 1, 2, and 3, which correspond to "never", "occasionally", "sometimes", and "often" respectively. The total scores of PSQI are composed of 7 domains: subjective sleep quality, sleep latency, sleep duration, habitual sleep efficiency, sleep disturbances, use of sleeping medication, and daytime dysfunction. In this study, according to whether the PSQI scores were greater than 5, sleep quality was defined as "good" (PSQI ≤ 5) or "poor" (PSQI > 5) [2, 3].

**Neuropsychiatric Inventory (NPI)**

NPI was used to evaluate neuropsychiatric symptoms of the participants. NPI surveys 12 different domains: delusion, hallucination, agitation, depression, anxiety, euphoria, apathy, disinhibition, aberrant motor disorder (AMD), sleep disorder, and appetite and eating disorder (AED). The NPI is based on information from informed caregivers. Two separated ratings were acquired for each domain: (1) frequency was rated on a 4 point scale from 0 to 4 (0= none, 1= occasional, 2= often, 3= most of time, 4= very frequent); (2) severity on a 3 point scale from 0 to 3 (0= none, 1= mild, 2= moderate, 3=severe), the score for each item is equal to frequency multiplied by severity. And total NPI score is calculated by adding all 12 items for a maximum total score of 144. The caregiver distress (NPI-D) was rated on a five point scale from 0 to 5 for each NPI domain (0= no distress, 1= minimal, 2= mild, 3= moderate, 4= moderately severe, 5= very severe or extreme), and the final sore of NPI-D is the sum of 12 items [4-6].

**Relative Stress Scale (RSS)**

RSS was used to evaluate the burden of caregivers. 15 items covering three aspects of burden from caregivers were accessed by this scale, including personal distress, life upset and negative feelings caused by the patients. The rating of RSS contains three levels: 0 = the least stress (never), 1 = the moderately stress (sometimes), 3 = the most stress (a great deal of time) with minimal total score of 0 and maximal of 30. The total score of these questions represented the stress that caregivers bear in caring for patients, so high score meant heavy burden of care [7].

**Patient Health Questionnaire-9 (PHQ-9)**

PHQ-9 was used to evaluate the depression status of the caregivers. The scale contains 9 items, and the score of each item can be divided into four levels of 0, 1, 2, and 3, which correspond to "never", "sometimes", "often", and "everyday" respectively. In our study, caregivers were defined as no depression (0-4), mild depression (5-9), moderate depression (10-14), moderately severe depression(15-19) and severe depression (20-27) [8].

**Generalized Anxiety Disorder Scale (GAD-7)**

GAD-7 was used to evaluate the anxiety status of the caregivers. The scale contains 7 items, and score of each item can be divided into four levels of 0, 1, 2, and 3, which correspond to "never", "sometimes", "often", and "everyday" respectively. In our study, caregivers were defined as no anxiety (0-4), mild anxiety (5-9), moderate anxiety (10-14) and severe anxiety (15-21) [9].

**References**

1 . Rubenstein LZ, Harker JO, Salva A, Guigoz Y, Vellas B (2001) Screening for undernutrition in geriatric

practice: Developing the Short-Form Mini-Nutritional Assessment (MNA-SF). J Gerontol A Biol Sci Med

Sci 56, M366-372. https://doi.org/10.1093/gerona/56.6.m366.

2 . Buysse DJ, Reynolds CF, 3rd, Monk TH, Berman SR, Kupfer DJ (1989) The Pittsburgh sleep quality index: A new instrument for psychiatric practice and research. Psychiatry Res 28, 193-213. https://doi.org/10.1016/0165-1781(89)90047-4.

3 . Dunleavy G, Bajpai R, Tonon AC, Chua AP, Cheung KL, Soh CK, Christopoulos G, Vries H, Car J (2019) Examining the factor structure of the Pittsburgh Sleep Quality Index in a multi-ethnic working population in Singapore. Int J Environ Res Public Health 16, 4590. https://doi.org/[10.3390/ijerph16234590](https://doi.org/10.3390/ijerph16234590)

4 . Cummings JL, Mega M, Gray K, Rosenberg-Thompson S, Carusi DA, Gornbein J (1994) The

Neuropsychiatric Inventory: Comprehensive assessment of psychopathology in dementia. Neurology

44, 2308-2314. https://doi.org/10.1212/wnl.44.12.2308.

5 . Cummings JL (1997) The Neuropsychiatric Inventory: Assessing psychopathology in dementia

patients. Neurology 48, S10-16. https://doi.org/10.1212/wnl.44.12.2308.

6 . Kaufer DI, Cummings JL, Christine D, Bray T, Castellon S, Masterman D, MacMillan A, Ketchel P,

DeKosky ST (1998) Assessing the impact of neuropsychiatric symptoms in Alzheimer's Disease: The

Neuropsychiatric Inventory Caregiver Distress Scale. J Am Geriatr Soc 46, 210-215.

https://doi.org/10.1002/gps.5134. Epub 2019 May 10.

7 . Eagles JM, Craig A, Rawlinson F, Restall DB, Beattie JA, Besson JA (1987) The psychological

well-being of supporters of the demented elderly. Br J Psychiatry 150, 293-298.

https://doi.org/10.1016/j.jalz.2015.04.011.

8 . Kroenke K, Spitzer RL, Williams JB (2001) The PHQ-9: validity of a brief depression severity

measure. J Gen Intern Med 16, 606-613. https://doi.org/[10.1046/j.1525-1497.2001.016009606.x](https://doi.org/10.1046/j.1525-1497.2001.016009606.x)

9 . Spitzer RL, Kroenke K, Williams JB, Löwe B (2006) A brief measure for assessing generalized anxiety

disorder: the GAD-7. Arch Intern Med 166, 1092-1097. https://doi.org/10.1001/archinte.166.10.1092

**2 Supplementary Figures and Tables**

## 2.1 Supplementary Figures


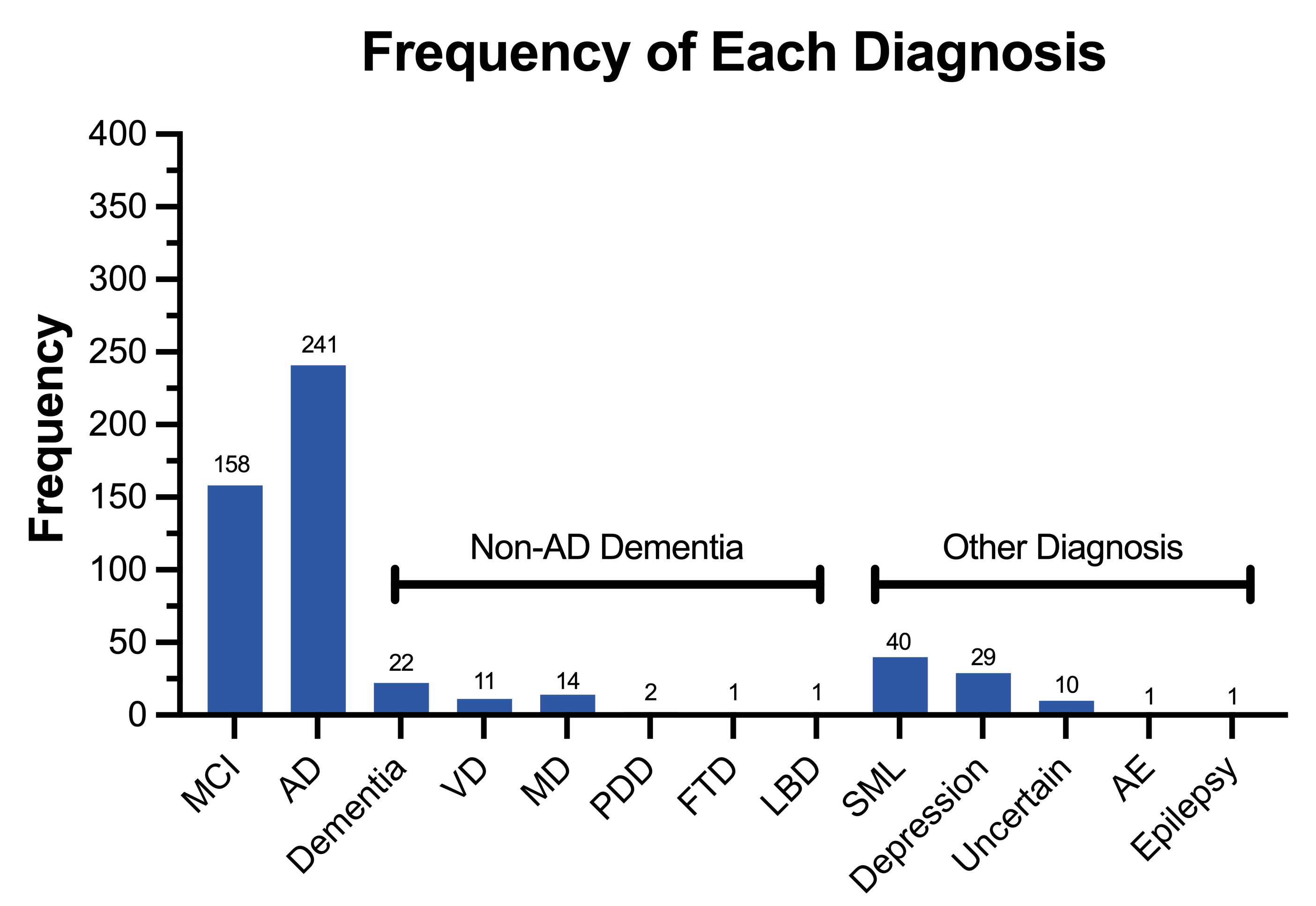


**Supplementary Figure S1. Frequency and classification of each diagnosis**

Of all 531 participants, 158 were diagnosed as MCI and AD took up the biggest proportion. Patients diagnosed as dementia, vascular dementia (VD), mixed dementia (MD), Parkinson’s Disease dementia (PDD), frontotemporal dementia (FTD), and Lewy Body dementia (LBD) were grouped as Non-AD Dementia group, and patients diagnosed as subjective memory loss (SML), depression, alcoholic encephalopathy (AE), and epilepsy as Other Diagnosis.


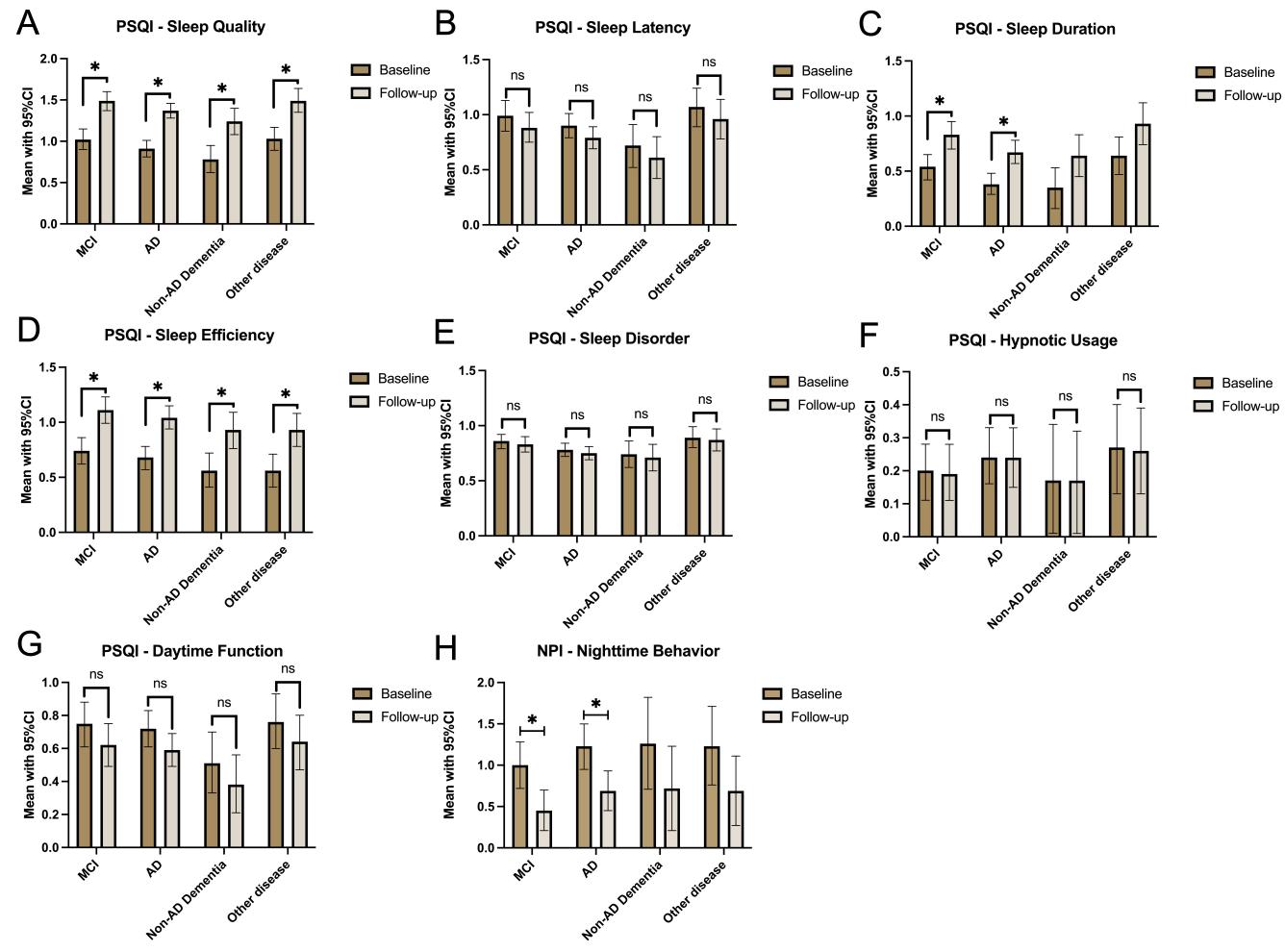


**Supplementary Figures 2. Generalized linear models for subitems of PSQI and nighttime behavior of NPI in each group.**

Generalized linear models for subitems of PSQI **(A-G)** and nighttime behavior of NPI **(H)**. **(A)** The sleep quality of patients from different diagnostic group became worse at follow-up than that at baseline (all *P*<0.05). **(B)** The sleep latency revealed no significant difference among all groups. **(C)** The sleep duration of patients with MCI and AD were statistically shortened at follow-up, compared to baseline performance(both *P*<0.05). **(D)** The sleep efficiency of patients from all groups worsened at follow-up (all *P*<0.05). **(E-G)** The sleep disorder, hypnotic usage, and daytime function of all patients showed no significant difference among all groups. **(H)** According to NPI, compared to baseline situation, MCI and AD patients revealed significant alleviation in nighttime behavior at follow-up (both *P*<0.05).


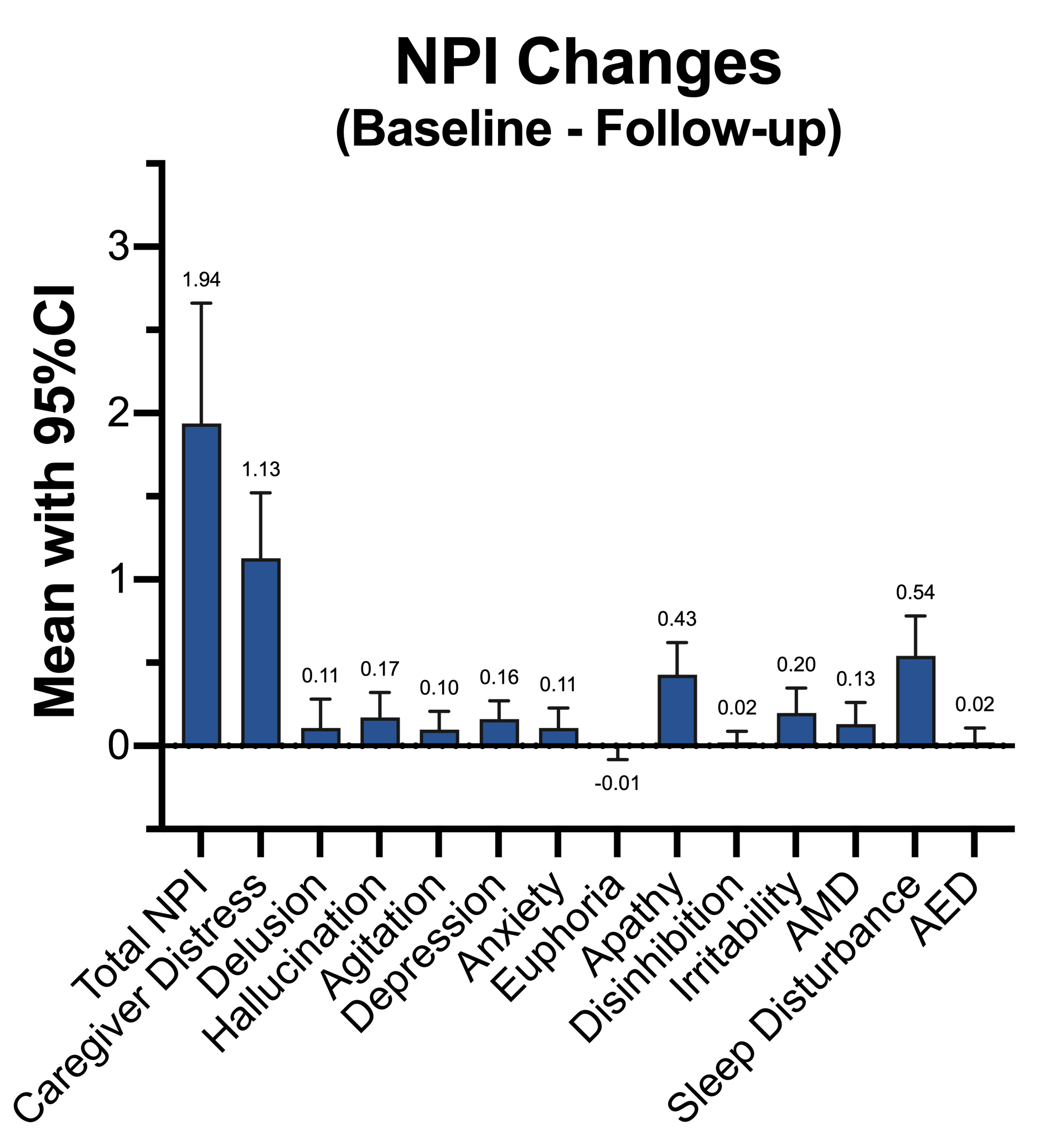


**Supplementary Figure** **3. Changes of NPI performance.**

The changes of performance in NPI and its subdomains were presented as mean with 95%CI. In general, nearly all NPS, except for euphoria, displayed a decline tendency in the follow-up period. For total NPI, it reduced by 1.94 points, and among the 12 subdomains, patients performance in sleep disturbance and apathy improved the most, by an average of 0.54 and 0.43 respectively.

## 2.1 Supplementary Tables

**Supplementary Table 1.Appetite and Weight change of the study population according to categories of nutritional status**

| Total (n=531) | Nutritional status | | | |
| --- | --- | --- | --- | --- |
|  |  |  |  |  |
|  | Malnutrition n=66 | At risk of malnutrition n=229 | Well-nourished n=236 | P |
|  |  |  |  |  |
| 427(80.4) | 13(19.7) | 184(80.3) | 230(97.5) | 0.000* |
| 45(8.5) | 17(25.8) | 27(11.8) | 1(0.4) |  |
| 59(11.1) | 36(54.5) | 18(7.9) | 5(2.1) |  |
|  |  |  |  |  |
| 355(66.9) | 4(6.1） | 134(58.5) | 217(91.9) | 0.000* |
| 41(7.1) | 20(30.3) | 21(9.2) | 0(0.0) |  |
| 29(5.5) | 4(6.1) | 8(3.5) | 17(7.2) |  |
| 106(20.0) | 38(57.6) | 66(28.8) | 2(0.8) |  |

531 cases enrolled and 8.5% of the participants stated loss of appetite, whereas 11.1% showed an obvious increase in appetite. Compared with other two categories, malnutrition displayed highest rate of increased food intake by 54.5%.

**Supplementary Table 2. Prevalence of NPS of Each Diagnostic Group at Baseline (%).**

| Parameters | All subjects |
| --- | --- |
| Food intake |  |
| Severe decrease | 13(2.4%） |
| Moderate decrease | 32(6.0%) |
| Severe increase | 53(10.0%) |
| Moderate increase | 6(1.1%) |
| No change | 427(80.4%) |
| Weight change |  |
| Weight loss ＞3kg | 21(4.0%) |
| Weight loss 1-3kg | 20(3.8%) |
| Weight gain ＞3kg | 2(0.4%) |
| Weight gain 1-3kg | 27(5.1%) |
| Does not Know | 106(20.0%) |
| No change | 355(66.9%) |
| Nutritional status |  |
| Malnutrition | 66(12.4%) |
| At risk of malnutrition | 229(43.1%) |
| Well-nourished | 236(44.4%) |

**Supplementary Table 3. Prevalence of NPS of Each Diagnostic Group at Baseline (%).**

|  | MCI | AD | Non-AD Dementia | Other Diagnosis | Total |
| --- | --- | --- | --- | --- | --- |
| NPI≥1 | 12.62 | 30.89 | 3.01 | 7.72 | 54.24 |
| Caregiver Distress | 9.98 | 27.31 | 1.69 | 5.84 | 44.82 |
| Delusion | 2.45 | 13.18 | 1.13 | 1.51 | 18.27 |
| Hallucination | 2.45 | 11.49 | 1.69 | 1.13 | 16.76 |
| Agitation | 3.01 | 6.59 | 0.94 | 0.56 | 11.10 |
| Depression | 3.39 | 6.21 | 0.94 | 3.58 | 14.12 |
| Anxiety | 4.52 | 5.84 | 0.94 | 2.82 | 14.12 |
| Euphoria | 0 | 1.13 | 0.38 | 0.19 | 1.70 |
| Apathy | 2.82 | 14.31 | 0.94 | 1.88 | 19.95 |
| Disinhibition | 0.94 | 3.77 | 0.56 | 0.75 | 6.02 |
| Irritability | 3.95 | 13.75 | 2.45 | 2.07 | 22.22 |
| AMD | 1.51 | 8.29 | 2.45 | 0.75 | 13.00 |
| Nighttime Behavior | 5.08 | 12.43 | 2.64 | 3.39 | 23.54 |
| AED | 0.38 | 2.26 | 0.19 | 0.38 | 3.21 |

**Supplementary Table 4. Prevalence of NPS of Each Diagnostic Group at Follow-up (%).**

|  | MCI | AD | Non-AD Dementia | Other Diagnosis | Total |
| --- | --- | --- | --- | --- | --- |
| NPI≥1 | 10.18 | 23.56 | 4.52 | 0.56 | 38.82 |
| Caregiver Distress | 8.12 | 20.70 | 4.61 | 4.29 | 37.72 |
| Delusion | 2.08 | 11.48 | 2.26 | 1.13 | 16.95 |
| Hallucination | 1.87 | 7.72 | 1.32 | 1.13 | 12.03 |
| Agitation | 1.87 | 4.31 | 1.69 | 0.56 | 8.44 |
| Depression | 2.83 | 4.31 | 0.57 | 1.31 | 9.02 |
| Anxiety | 2.83 | 4.90 | 0.57 | 1.88 | 10.17 |
| Euphoria | 0.57 | 0.95 | 0.19 | 0.38 | 2.09 |
| Apathy | 2.26 | 6.99 | 1.13 | 1.13 | 11.51 |
| Disinhibition | 0.95 | 2.27 | 0.37 | 0.56 | 4.16 |
| Irritability | 2.83 | 10.17 | 2.26 | 2.07 | 17.32 |
| AMD | 2.08 | 5.85 | 0.94 | 0.18 | 9.06 |
| Nighttime Behavior | 3.96 | 7.90 | 1.51 | 2.26 | 15.62 |
| AED | 0.57 | 2.81 | 0.19 | 0.56 | 4.14 |

**Supplementary Table 5. Changes of NPS Prevalence of Each Diagnostic Group (%).**

| Follow-up - Baseline | MCI | AD | Non-AD Dementia | Other Diagnosis | Total |
| --- | --- | --- | --- | --- | --- |
| NPI≥1 | -2.44 | -7.33 | 1.51 | -7.16 | -15.42 |
| Caregiver Distress | -1.86 | -6.61 | 2.92 | -1.55 | -7.10 |
| Delusion | -0.37 | -1.70 | 1.13 | -0.38 | -1.32 |
| Hallucination | -0.58 | -3.77 | -0.37 | 0.00 | -4.73 |
| Agitation | -1.14 | -2.28 | 0.75 | 0.00 | -2.66 |
| Depression | -0.56 | -1.90 | -0.37 | -2.27 | -5.10 |
| Anxiety | -1.69 | -0.94 | -0.37 | -0.94 | -3.95 |
| Euphoria | 0.57 | -0.18 | -0.19 | 0.19 | 0.39 |
| Apathy | -0.56 | -7.32 | 0.19 | -0.75 | -8.44 |
| Disinhibition | 0.01 | -1.50 | -0.19 | -0.19 | -1.86 |
| Irritability | -1.12 | -3.58 | -0.19 | 0.00 | -4.90 |
| AMD | 0.57 | -2.44 | -1.51 | -0.57 | -3.94 |
| Nighttime Behavior | -1.12 | -4.53 | -1.13 | -1.13 | -7.92 |
| AED | 0.19 | 0.55 | 0.00 | 0.18 | 0.93 |

**Supplementary Table 6. Generalized estimation equation for mixed effects on each subitems of PSQI.**

| ***Item*** | **Time** | **B** | **Wald Chi-Square Test** | **P Value** |
| --- | --- | --- | --- | --- |
| *Sleep Quality* | Baseline | 0^a^ | - | - |
|  | Follow-up | 0.461 | 89.250 | < 0.001 |
| *Sleep Latency* | Baseline | 0^a^ | - | - |
|  | Follow-up | - 0.109 | 3.569 | 0.059 |
| *Sleep Duration* | Baseline | 0^a^ | - | - |
|  | Follow-up | 0.291 | 24.017 | < 0.001 |
| *Sleep Efficiency* | Baseline | 0^a^ | - | - |
|  | Follow-up | - 0.256 | 0.168 | 0.682 |
| *Sleep Disorder* | Baseline | 0^a^ | - | - |
|  | Follow-up | - 0.026 | 0.670 | 0.413 |
| *Hypnotic Usage* | Baseline | 0^a^ | - | - |
|  | Follow-up | - 0.006 | 0.019 | 0.889 |
| *Daytime Function* | Baseline | 0^a^ | - | - |
|  | Follow-up | -0.128 | 7.647 | 0.006 |

Model: (Intercept) Time. a: Set to zero because this parameter is redundant.

The Generalized Linear Models were performed to all PSQI subdomains with intercept of time of investigation. For all patients, the sleep quality and duration were significantly worsened (both *P*<0.001) while the daytime function became better (*P*=0.006).
